# New and Cytotoxic Components from *Antrodia camphorata*

**DOI:** 10.3390/molecules191221378

**Published:** 2014-12-19

**Authors:** Tzong-Huei Lee, Chien-Chih Chen, Jih-Jung Chen, Hui-Fen Liao, Hsun-Shuo Chang, Ping-Jyun Sung, Mei-Hwei Tseng, Sheng-Yang Wang, Horng-Huey Ko, Yueh-Hsiung Kuo

**Affiliations:** 1 Institute of Fisheries Science, National Taiwan University, Taipei 106, Taiwan; E-Mail: thlee1@ntu.edu.tw; 2 Department of Nursing, Hungkuang University, Taichung 443, Taiwan; E-Mail: ccchen@sunrise.hk.edu.tw; 3 Department of Biotechnology, Hungkuang University, Taichung 443, Taiwan; 4 Department of Pharmacy, Tajen University, Pingtung 907, Taiwan; E-Mail: jjchen@mail.tajen.edu.tw; 5 Department of Biochemical Science and Technology, National Chiayi University, Chiayi 600, Taiwan; E-Mail: liaohf@seed.net.tw; 6 School of Pharmacy, College of Pharmacy, Kaohsiung Medical University, Kaohsiung 807, Taiwan; E-Mail: hschang@kmu.edu.tw; 7 Graduate Institute of Natural Products, College of Pharmacy, Kaohsiung Medical University, Kaohsiung 807, Taiwan; 8 Graduate Institute of Marine Biotechnology, National Dong Hwa University, Pingtung 944, Taiwan; E-Mail: pjsung@nmmba.gov.tw; 9 Department of Life Science and Institute of Biotechnology, National Dong Hwa University, Pingtung 944, Taiwan; 10 National Museum of Marine Biology and Aquarium, Pingtung 944, Taiwan; 11 Department of Applied Physics and Chemistry, Taipei Municipal University of Education, Taipei 100, Taiwan; E-Mail: biomei@tmue.edu.tw; 12 Department of Forestry, National Chung-Hsing University, Taichung 402, Taiwan; E-Mail: taiwanfir@dragon.nchu.edu.tw; 13 Department of Fragrance and Cosmetic Science, College of Pharmacy, Kaohsiung Medical University, Kaohsiung 807, Taiwan; E-Mail: hhko@kmu.edu.tw; 14 Department of Chinese Pharmaceutical Sciences and Chinese Medicine Resources, China Medical University, Taichung 404, Taiwan; 15 Department of Biotechnology, Asia University, Taichung 413, Taiwan

**Keywords:** *Antrodia camphorata*, phenylmethanoid, dihydrostilbene, *abeo*-ergostane, cytotoxicity

## Abstract

The solid-state cultured products of *Antrodia camphorata* as health foods has been blooming for the past few decades in Taiwan. In continuing our studies on the chemical constituents of the solid-state cultured products of this fungus, 6-methoxy-4-methyl-2,3-(methylenedioxy)phenol (**1**) and 4,4'-(ethane-1,2-diyl)bis(2,3,6-trimethoxyphenol)(**2**) together with 2,3,6-trimethoxy-4-methylphenol (**3**), 1(10→6)*abeo*-ergosta-5,7,9,22-tetraen-3α-ol (**4**), citreoanthrasteroid B (**5**) and dankasterones A (**6**) and B (**7**) were purified by a series of column chromatography. Their structures were elucidated by spectral data analysis. For bioactivity assay, compounds **4**–**7** showed significant cytotoxicity toward murine colorectal CT26 and human leukemia K562 cancer cell lines with IC_50_ values ranging from 6.7 to 15.3 µM and from 12.5 to 23.1 µM, respectively.

## 1. Introduction

**Figure 1:**
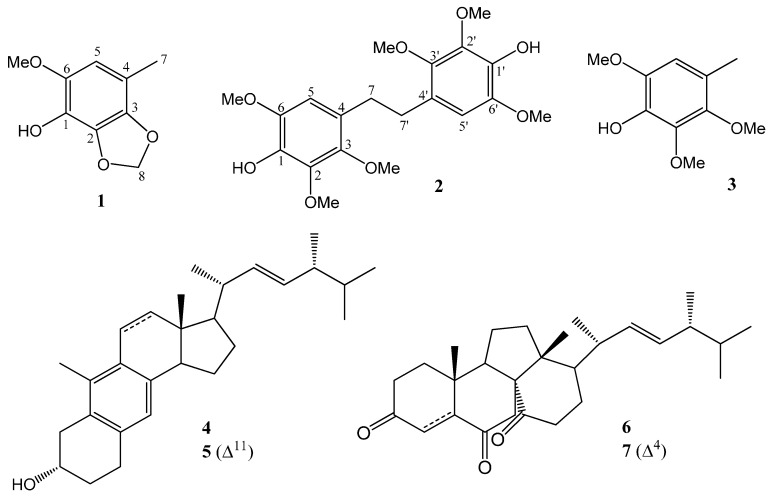
Chemical structures of **1**–**7** isolated in this study.

*Antrodia camphorata* Wu, Ryvarden & Chang (Polyporaceae), a fungus indigenous to Taiwan, was used by the aborigine as a hepatinica and anti-alcoholic agent initially and has been gradually used as a folk remedy for the treatment of liver cancer and various cardiovascular diseases in the past few decades [1]. The fermentation and development of this fungus have already become one of the major components of the biotechnology industry in Taiwan recently, and some of the chemical entities isolated from the fermented products, e.g., antroquinonol [2] and ergosta-7,9,22*E*-trien-3β-ol [3], were selected as leads for new drug development. Although over one hundred compounds have been identified from this fungus so far [4,5,6], new chemical entities are still reported continually by virtue of varied culturing conditions for this fungus. In continuing our investigations on the chemical constituents of the mycelium of *Antrodia camphorata*, one new phenylmethanoid, 6-methoxy-4-methyl-2,3-(methylenedioxy)phenol (**1**), and one new stilbene, 4,4'-(ethane-1,2-diyl)bis(2,3,6-trimethoxyphenol)(**2**), together with 2,3,6-trimethoxy-4-methylphenol (**3**), 1(10→6)*abeo*-ergosta-5,7,9,22-tetraen-3α-ol (**4**), citreoanthrasteroid B (**5**) and dankasterones A (**6**) and B (**7**) (Figure 1) were isolated and characterized on the basis of the spectral analysis. The paper describes the isolation and identification of compounds **1**–**7** from *A.* *camphorata* along with their anticancer effects in murine colorectal cancer CT26 cells and human leukemia K562 cells.

## 2. Results and Discussion

From the methanolic extracts of the solid-state cultured, fermented dried powder of *A. camphorata,* seven major compounds, including one new phenylmethanoid, **1**, and one new stilbene, **2**, along with a known phenylmethanoid, **3**, and four steroids, **4**–**7**, were isolated by sequential separation on Si-gravity column and normal-phase HPLC. Compound **3** was obtained as an amorphous white solid whose ^1^H, ^13^C-NMR, IR, optical rotation and MS were consistent with those of synthetic 2,3,6-trimethoxy-4-methylphenol [7], and this is the first time Compound **3** has been isolated from a natural resource. Compounds **4** and **5**, two rare 1(10→6)*abeo*-ergostane-type steroids, were identified as 1(10→6)*abeo*-ergosta-5,7,9,22-tetraen-3α-ol (**4**), obtained previously from the stromata of *Epichloe typhina* [8], and citreoanthrasteroid B (**5**), isolated from a hybrid bacterial strain, KO 0231 [9]. Compounds **6** and **7**, two uncommon 13(14→8)*abeo*-ergostane-type steroids, were characterized as respective dankasterones A and B, which were isolated previously from a *Halichondria* sponge-derived fungus, *Gymnascella dankaliensis* [10].

**Figure 2:**
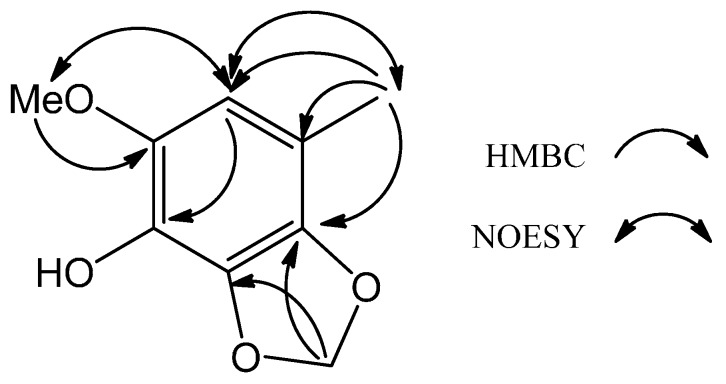
Key HMBC and NOESY of **1**.

Compound **1** was afforded as a colorless amorphous solid with the molecular formula of C_9_H_10_O_4_ as established through the analysis of its ^13^C-NMR and HRESIMS data. The IR absorption peaks of **1** at 3277, 1626 and 1527 cm^−1^ indicated that **1** contained a benzene moiety bearing a hydroxy functionality as reflected in its ^1^H- and ^13^C-NMR data. The ^1^H-NMR of **1** exhibited a phenyl proton at δ_H_ 6.27 (s, 1H), a benzene-borne methyl at δ_H_ 2.16 (s, 3H), a benzene-borne methoxyl at δ_H_ 3.82 (s, 3H) and a methylenedioxy group at δ_H_ 5.91 (s, 2H), which were further confirmed by their corresponding six phenyl resonances at δ_C_ 109.0 (d), 118.9 (s), 131.9 (s), 133.6 (s), 135.0 (s) and 136.8 (s), one methyl resonance at δ_C_ 15.8 (q), one methoxyl resonance at δ_C_ 57.2 (q) and one dioxygenated methylene resonance at δ_C_ 101.5 (t) in the ^13^C-NMR of **1**. Further analysis of the 2D NMR data of **1**, key cross-peaks of δ_H_ 2.16 (H_3_-7)/δ_C_ 109.0 (C-5), 118.9 (C-4) and 131.9 (C-3), δ_H_ 3.82 (OMe-6)/δ_C_ 136.8 (C-6), δ_H_ 5.91 (H_2_-8)/δ_C_ 131.9 (C-3) and 135.0 (C-2), δ_H_ 6.27 (H-5)/δ_C_ 133.6 (C-1) in the HMBC spectrum and mutual-correlated cross peaks of δ_H_ 6.27 (H-5)/δ_H_ 3.82 (OMe-6) and δ_H_ 2.16 (H_3_-7) in the NOESY spectrum (Figure 2) established the locations of all of the functional groups attached to the benzene ring. Thus, **1** was deduced as the shown phenylmethanoid and was named 6-methoxy-4-methyl-2,3-(methylenedioxy)phenol.

**Figure 3:**
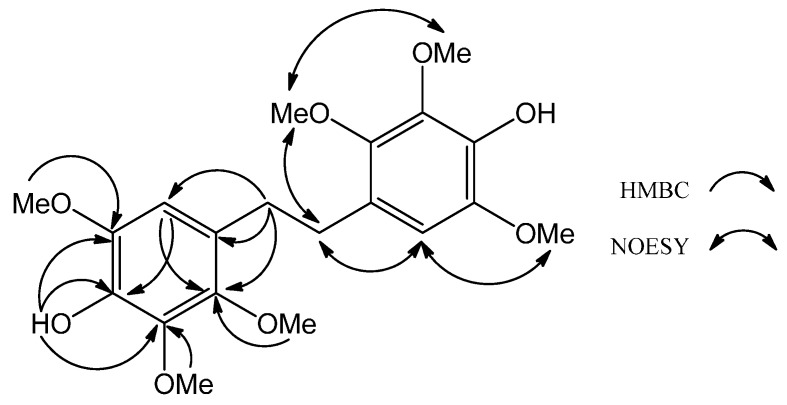
Key HMBC and NOESY of **2**.

Compound **2**, obtained as a colorless amorphous solid, was a symmetrical chemical entity as judged from its molecular formula, C_20_H_26_O_8_, deduced from HRESIMS and only ten resonances in the ^13^C-NMR spectrum. The IR spectrum of **2** confirmed the presence of a hydroxyl group (3410 cm^−1^) and a benzene ring (1610 and 1503 cm^−1^). The ^13^C-NMR along with the DEPT of **2** displayed only ten signals (indeed, twenty signals due to symmetry), including one methylene carbon at δ_C_ 31.4, three benzene-borne methoxyl carbons at δ_C_ 56.4, 60.7 and 61.0 and six phenyl carbons at δ_C_ 107.2, 125.2, 137.3, 140.4, 143.2 and 145.2. The ^1^H-NMR spectrum showed signals for the methylene group at δ_H_ 2.78 (s, 2H), three methoxyl groups at δ_H_ 3.79 (s, 3H), 3.80 (s, 3H) and 3.91 (s, 3H), one hydroxyl group at δ_H_ 5.45 (brs, 1H) and one phenyl proton at δ_H_ 6.38 (s, 1H). The above assignments indicated that **2** had a stilbene skeleton with three methoxyl and one hydroxyl groups on each benzene ring. The locations of three methoxy, one hydroxy and one phenyl proton were further corroborated by key HMBC interpretations, including δ_H_ 2.78 (H-7, -7')/δ_C_ 107.2 (C-5, -5'), 125.2 (C-4, -4') and 145.2 (C-3, -3'), δ_H_ 3.79 (OMe-3, -3')/δ_C_ 145.2 (C-3, -3'), δ_H_ 3.80 (OMe-6, -6')/δ_C_ 143.2 (C-6, -6'), δ_H_ 3.91 (OMe-2, -2')/δ_C_ 140.4 (C-2, -2'), δ_H_ 5.45 (OH-1, -1')/δ_C_ 137.3 (C-1, -1'), 140.3 (C-2, -2'), 143.2 (C-6, -6') and δ_H_ 6.38 (H-5, -5')/δ_C_ 137.3 (C-1, -1') and 145.2 (C-3, -3'), as well as key NOESY correlations, including δ_H_ 6.38 (H-5, -5')/δ_H_ 2.78 (H-7, -7') and 3.80 (OMe-6, -6') and δ_H_ 3.79 (OMe-3, -3')/δ_H_ 2.78 (H-7, -7') and 3.91 (OMe-2, -2') (Figure 3). Accordingly, **1** was determined as the shown dihydrostilbene and was named 4,4′-(ethane-1,2-diyl)bis(2,3,6-trimethoxyphenol). Compound **2** was speculated from the oxidative coupling between two molecules of Compound **3**.

For the anticancer activity, Table 1 shows the IC_50_ values of compounds **1**‒**7** against murine colorectal cancer CT26 cells and human leukemia K562 cells. Compounds **1**‒**3** exhibited no obvious effect toward CT26 and K562 cells with their IC_50_ values higher than 20 μM, and **4**–**7** showed significant cytotoxicity toward murine colorectal CT26 and human leukemia K562 cancer cell lines with IC_50_ values ranging from 6.7 to 18.2 μM and from 12.5 to 23.1 μM, respectively. At the same condition, the IC_50_ value of staurosporine against K562 leukemia cells was 16.7 nM.

**Table 1:** IC_50_ values of compounds **1**–**7** against colorectal cancer CT26 and leukemia K562 cells.

Compounds	IC_50_ (μM)	
	CT26	K562
**1**	>20	>20
**2**	>20	>20
**3**	>20	>20
**4**	15.3	19.9
**5**	18.2	12.5
**6**	6.7	>20
**7**	8.4	23.1

The IC_50_ value of staurosporine, the positive control, against K562 cells was 16.7 nM.

## 3. Experimental

### 3.1. General

Optical rotations were measured on a JASCO DIP-1000 polarimeter (Tokyo, Japan). ^1^H and ^13^C-NMR were acquired on a Bruker DMX-500 (Ettlingen, Germany). Low resolution and high resolution mass spectra were obtained using an API4000 triple quadrupole mass spectrometer (Applied Biosystems, Foster City, CA, USA) and a Synapt High Definition Mass Spectrometry system with an ESI interface and a TOF analyzer (Waters Corp., Manchester, UK), respectively. IR spectra were recorded on a JASCO FT/IR 4100 spectrometer (Tokyo, Japan). TLC was performed using silica gel 60 F_254_ plates (200 µm, Merck, Taipei, Taiwan). 

### 3.2. Fungal Material

Freeze-dried powder of *Antrodia camphorata* was provided by Kang Jian Biotech Corp. Ltd, Nantan, Taiwan R.O.C.

### 3.3. Extraction and Isolation

The dried powder (2.83 kg) of solid-state cultured *Antrodia camphorata* was extracted with 12 L methanol for three times, and the concentrated residues (323.8 g) were suspended in H_2_O and further partitioned three times with equal volumes of ethyl acetate, then concentrated in vacuum to dryness (131.5 g). The ethyl acetate extract was applied onto an open column with silica gel. The column was eluted with mixtures of *n*-hexane, ethyl acetate and methanol, and each 1 L was collected as one fraction. Fractions 16–33 were combined and evaporated to dryness (1.4 g), which were further purified by HPLC on a semi-preparative Phenomenex Luna Si column (5 µm, 10 × 250 mm) with *n*-hexane–ethyl acetate (90:10, *v*/*v*) as the eluent, 2 mL/min, obtaining **1** (4.6 mg), **2** (6.4 mg) and **3** (8.9 mg). Fractions 92–124 were combined and evaporated to dryness (33.1 g), which were further purified by HPLC on the same column with *n*-hexane–ethyl acetate (50:50, *v*/*v*) as the eluent, 2 mL/min, obtaining **4** (5.6 mg), **5** (7.4 mg), **6** (10.3) and **7** (9.2 mg).

*6-Methoxy-4-methyl-2,3-(methylenedioxy)phenol* (**1**): Colorless amorphous solid; IR (neat): ν_max_ 3277, 3015, 1626, 1527, 1440, 1341, 1208, 1129, 1043, 925 cm^−1^. ^1^H-NMR (CDCl_3_, 400 MHz): δ_H_ 2.16 (3H, s, H-7), 3.82 (3H, s, OMe-6), 5.91 (2H, s, H-8), 6.27 (1H, s, H-6). ^13^C-NMR (CDCl_3_, 100 MHz): δ_C_ 15.8 (C-7), 57.2 (Ome-6), 101.5 (C-8), 109.0 (C-5), 118.9 (C-4), 131.9 (C-3), 133.6 (C-1), 135.0 (C-2), 136.8 (C-6). ESIMS: *m*/*z* = 183 [M+H]^+^. HRESIMS: *m*/*z* = 183.0653 [M+H]^+^ (calcd. for C_9_H_11_O_4_, 183.0657).

*4,4'-(Ethane-1,2-diyl)bis(2,3,6-trimethoxyphenol)* (**2**): Colorless amorphous solid; IR (neat): ν_max_ 3410, 3012, 2956, 2860, 1610, 1503, 1420, 1325, 1248, 1198, 1125, 1076, 970, 880. ^1^H-NMR (CDCl_3_, 400 MHz): δ_H_ 2.78 (4H, s, H-7, -7'), 3.79 (6H, s, OMe-3, -3'), 3.80 (6H, s, OMe-6, -6'), 3.91 (6H, s, OMe-2, -2'), 5.45 (2H, brs, OH-1, -1'), 6.38 (2H, s, H-5, -5'). ^13^C-NMR (CDCl_3_, 100 MHz): δ_C_ 31.4 (C-7, -7'), 56.4 (OMe-3, -3'), 60.7 (OMe-6, -6'), 61.0 (OMe-2, -2'), 107.2 (C-5, -5'), 125.2 (C-4, -4'), 137.3 (C-1, -1'), 140.4 (C-2, -2'), 143.2 (C-6, -6'), 145.2 (C-3, -3'). ESIMS: *m*/*z* = 395 [M+H]^+^. HRESIMS: *m*/*z* = 395.1710 [M+H]^+^ (calcd. for C_20_H_27_O_8_, 395.1706).

### 3.4. Cells and Viability Assay

Murine colorectal cancer CT26 cells were cultured in RPMI1640 medium (Gibco, Grand Island, NY, USA) supplemented with 10% heat-inactivated fetal bovine serum (FBS; Hyclone, Logan, UT) and 2 mM l-glutamine at 37 °C in a humidified 5% CO_2_ incubator. The viability of CT26 cells was determined by using the 3-(4,5-dimethylthiazol-2-yl)-2,5-diphenyltetrazolium bromide (MTT, Sigma) colorimetric assay. Human chronic myeloid leukemia K562 cells were cultured in RPMI 1640 medium, 10% FBS and 2 mM l-glutamine at 37 °C in an incubator. The viability of K562 cells with compound treatments for one day were measured using the Trypan blue dye exclusion test. The IC_50_ values of compounds in CT26 and K562 cells were determined by using SigmaStat software (Jandel Scientific, San Rafael, CA, USA).

## 4. Conclusions

In this study, three C_6_-C_1_ derivatives, as well as four phytosteroids with rearranged skeletons were isolated from the industrial fermented products of *Antrodia camphorata*. Of the compounds isolated, four phytosteroids exhibited significant cytotoxicity against cancer cell lines, which may, to some extent, account for the traditional uses of this fungal product as a health food to treat cancer. More experiments should be performed to deduce the action modes of these compounds.
